# New Advances on Pathophysiology of Diabetes Neuropathy and Pain Management: Potential Role of Melatonin and DPP-4 Inhibitors

**DOI:** 10.3389/fphar.2022.864088

**Published:** 2022-04-12

**Authors:** Prabhakar Busa, Yaswanth Kuthati, Niancih Huang, Chih-Shung Wong

**Affiliations:** ^1^ Department of Anesthesiology, Cathay General Hospital, Taipei, Taiwan; ^2^ Department of Anesthesiology, Tri-Service General Hospital, Taipei, Taiwan; ^3^ Grauate Institute of Medical Sciences, National Defense Medical Center, Taipei, Taiwan

**Keywords:** diabetes mellitus (DM), neuropathic pain, neuroinflammation, melatonin, redox levels, antioxidant, anti-inflammatory

## Abstract

Pre-diabetes and diabetes are growing threats to the modern world. Diabetes mellitus (DM) is associated with comorbidities such as hypertension (83.40%), obesity (90.49%), and dyslipidemia (93.43%), creating a substantial burden on patients and society. Reductive and oxidative (Redox) stress level imbalance and inflammation play an important role in DM progression. Various therapeutics have been investigated to treat these neuronal complications. Melatonin and dipeptidyl peptidase IV inhibitors (DPP-4i) are known to possess powerful antioxidant and anti-inflammatory properties and have garnered significant attention in the recent years. In this present review article, we have reviewed the recently published reports on the therapeutic efficiency of melatonin and DPP-4i in the treatment of DM. We summarized the efficacy of melatonin and DPP-4i in DM and associated complications of diabetic neuropathy (DNP) and neuropathic pain. Furthermore, we discussed the mechanisms of action and their efficacy in the alleviation of oxidative stress in DM.

## Introduction

**FIGURE 1:**
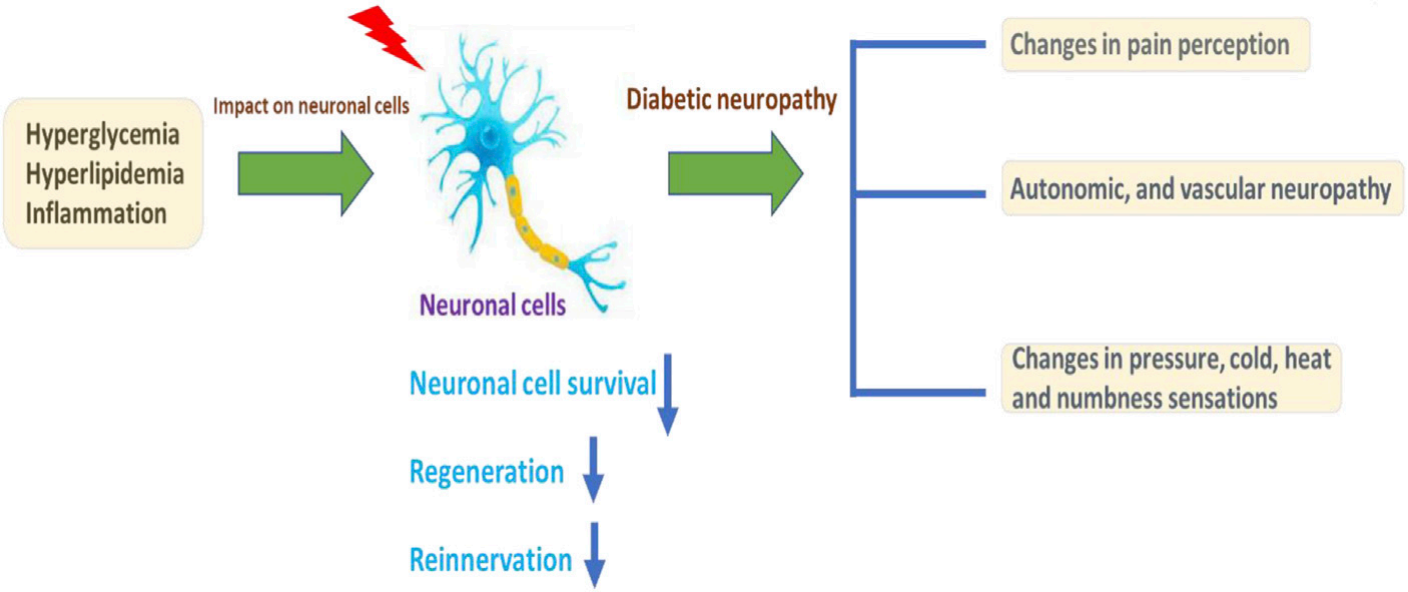
Illustration of DM conditions’ influence on the neuronal cells and DNP development, with further cellular consequences.

Diabetes mellitus (DM) is globally affecting major diseases, and it is a major death-causing disease in well-developed countries, according to the WHO survey (Corriere, Rooparinesingh, & Kalyani, 2013) (Sherr & Lipman, 2015). DM is characterized by a lack of production or cellular uptake of insulin, which depends on various factors (Ostenson, 2001) (S. Dong et al., 2019). In type 2 DM, it is characterized by less secretion of insulin to control the blood sugar in the body (Uno et al., 2018) (Porte, 2001; Reinehr, 2005). DM with other diseases becomes more complicated, and potential treatment procedures are needed to control progression (Harding, Pavkov, Magliano, Shaw, & Gregg, 2019; Pearce, Simo, Lovestam-Adrian, Wong, & Evans, 2019; Y. Zhao, Ye, Boye, Holcombe, & Swindle, 2009). Insulin is an essential enzyme to control glucose levels in the body (E. G. Hong et al., 2007). In the insulin resistance condition, more insulin levels are needed compared with normal conditions to overcome DM (Girgis, Scalley, & Park, 2012; Love et al., 2021; Pang & Narendran, 2008). Type 2 DM occurs in a genetic or acquired manner; in recent reports, authors have notified it is a complex and multifactorial metabolic disease (Smith-Palmer et al., 2014; Q. Q. Yang et al., 2021). Lack of insulin levels impacts the metabolism of carbohydrates, proteins, and lipids to develop hyperglycemia and hyperlipidemia conditions (Lodovici, Bigagli, Bardini, & Rotella, 2009; Sanz-Nogues, Mustafa, Burke, O'Brien, & Coleman, 2020; Y. Zhao et al., 2009). The hyperglycemia and hyperlipidemia conditions advanced complicated diseases such as neuropathy, retinopathy, cognitive defects, Parkinson’s, cardiomyopathy, atherosclerosis, and nephropathy. Type 2 DM is an evolving disease from metabolic disorders to inflammatory complications and further contributes to the development of neuropathic pain and neuropathy, as explained in Figure 1(Kuniss, Freyer, Muller, Kielstein, & Muller, 2019; Nijpels, Beulens, van der Heijden, & Elders, 2019). The long-term innate immune system activation in type 2 DM is due to chronic inflammation, according to recent reports (L. Li et al., 2016; Muggeo, 1998). In type 2 DM, insulin resistance and hyperglycemic conditions lead to the development of the neuronal complications (Cerveny, Leder, & Weart, 1998; Demir, Nawroth, Herzig, & Ustunel, 2021). The major cause of DM is peripheral nervous system (PNS) damage, a common type of nerve damage of the bilateral and symmetric nerves of the feet. The distal to proximal gradient of severity is known as stocking glove neuropathy, which is called DNP (Alam, 2020; Papanas, 2020). DNP is primarily a disorder of sensory nerves. In the early stages of DNP, patients experience positive sensory symptoms in their feet such as pain, tingling, and prickling sensations and negative symptoms such as numbness (P. Li, Yuan, & He, 2019; Maddaloni et al., 2021; Sima, 2008). DNP sensory management may induce pain when the feet are touched (allodynia) and increase the noxious stimuli (hyperglycemia) (Kahl, 2017; Aliabadi, Moradian, Rahmanian, & Mohammadi, 2021; Rumora et al., 2021). In the late stage of the disease, evidence of motor nerve dysfunction was observed. The fact that why sensory nerves are mainly vulnerable in DM is not clear until now compared with motor nerves (C. C. Chao, Tseng, & Hsieh, 2011; Falco et al., 2021; Yu, Zhao, Cao, Zhu, & Li, 2017; B. Q. Zhu, Quinn, & Fritschi, 2018).

Melatonin is a hormone secreted from the pineal gland. The plasma concentration of melatonin differs during the day (low) and night (high) (Rocha, Rato, Martins, Alves, & Oliveira, 2015). The plasma concentration of melatonin varies depending on various factors such as light, age, and sex. According to recent reports, the plasma level melatonin concentration (picomolar) is high in nocturnal animals (Cagnacci, 1996; Pohanka, 2013). Melatonin shows various pharmacological effects such as anti-inflammatory, antioxidant, neuroprotection, and inhibitory action in the central and peripheral nervous system (Beyer, Steketee, & Saphier, 1998; J. G.; Lee et al., 2019). These qualities suggest that it may be effective in the treatment of DNP (Xie, Fan, He, & Huang, 2020). Melatonin has low toxic effects, according to recent publications. High dose of 800 mg/kg in rats shows no adverse effects. The aim of this review is to provide an examination of previous reports reporting the actions of melatonin on DNP.

The DPP-4 enzyme rapidly degrades the blood plasma glucagon-like peptide-1 (GLP-1) and influences fasting glycemia in type 2 DM. Therefore, DPP-4i are known as potent antioxidant agents used for prolongation of GLP-1 half-life, increasing the GLP-1 levels and enhancing the secretion of insulin by inhibiting the DPP-4 enzyme to maintain glucose homeostasis. In this review, we focused on DPP-4i and its antioxidant properties for the treatment of DM and its complications.

**FIGURE 2:**
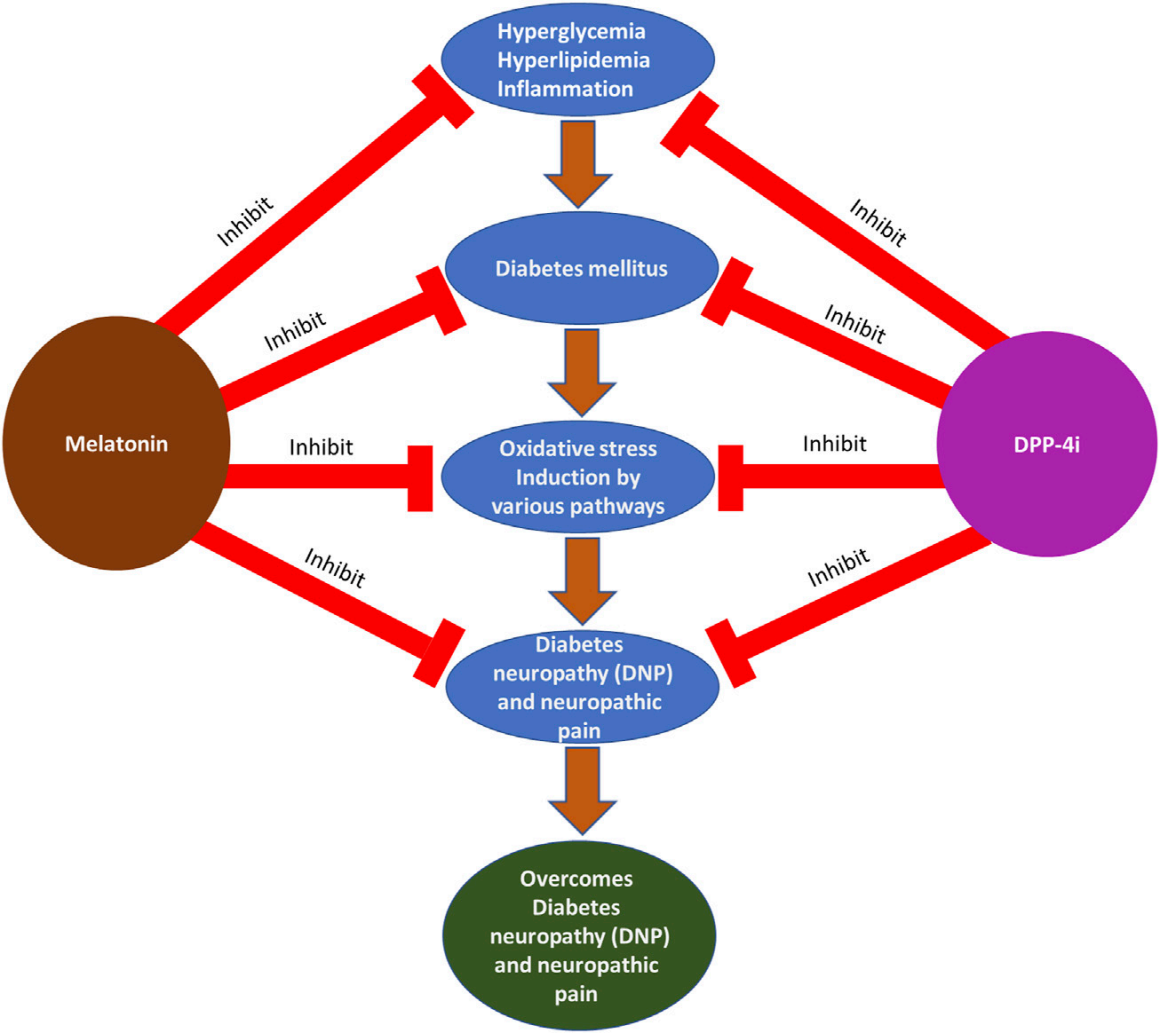
Photographical representation of the hypothesis of DM and its complications: treatments with melatonin and DPP-4i at various stages.

The rationale design of this narrative review mainly focuses on DM and its complications and treatment with melatonin and DPP-4i for DNP and neuropathic pain. Initially, we have focused on the oxidative stress induction pathways in DM that lead to DNP and neuropathic pain. Furthermore, we explained DM pathogenesis and neuropathic pain mechanisms. Finally, we explained the advantages of using melatonin and DPP-4i for the management of DM and its complications and their potent antioxidant properties. The hypothesis is described with a schematic diagram, as shown in Figure 2.

## Oxidative Stress Mechanistic Features in Diabetic Neuropathy

**FIGURE 3:**
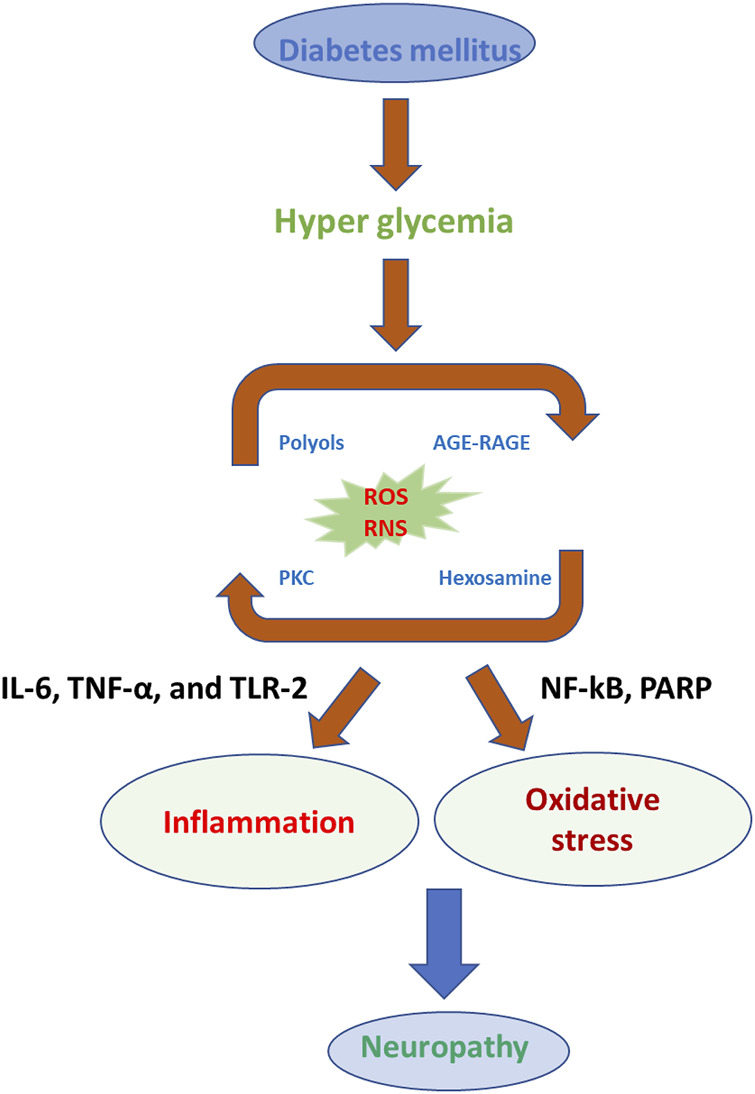
Photographical representation of oxidative stress consequences in the development of neuropathy in DM.

According to previous reports, low-grade and sub-clinical inflammation and pain depend on the risk factors for diabetes and insulin resistance in hyperglycemia (Figure 3) (Hey-Mogensen et al., 2010; Anthonsen, Larsen, Pedersen, Dalgaard, & Kvetny, 2013; Kassan et al., 2014; Manoharan et al., 2019). The reactive oxygen species (ROS) generation signals for neuropathy associated with DM complications (J. P. Chen, Xu, Liao, & Zhang, 2020; S. H. Lee et al., 2016; Nogueira-Machado et al., 2006; Sommese et al., 2018). The ROS-induced oxidative stress elevated the pro-inflammatory and neuropathic pain-related biomarkers in DM (Turecky, Kupcova, Uhlikova, & Mojto, 2014; Sharifzadeh, Ranjbar, Hosseini, & Khanavi, 2017; Liu et al., 2019). In another report, convincing arguments related to obese patients’ complications with type 2 DM; insulin resistance contributes to the generation and increasing the secretion of tumor necrosis factor–α (TNF-α), which participates in further consequences of neuropathic pain and degeneration of neurons (Ghouini, Rahal, & Djoghlaf, 2016; Z. H.; Yang & Peng, 2010; C. Y.; Zhao, Dai, & Zhou, 2016). The type 2 DM, ROS, and reactive nitrogen species (RNS) are collectively described as oxidative stress-inducing agents to imbalance the cellular redox environment to involve in the neuropathic pain and inflammation (Schofield & Sutherland, 2012; Fagundes-Netto, Anjos, Volpe, & Nogueira-Machado, 2013; Shi, Yang, & Jiang, 2016; Elzinga et al., 2019; Tian et al., 2021). The low and moderate levels of ROS and RNS are very useful for fighting against infections and maintaining cellular structural integrity. The *in vivo* and clinical studies demonstrate a promising relationship between ROS and neurological complications (Barutta et al., 2011; Mihanfar et al., 2021; Akbarzadeh, Darband, Sadighparvar, and Majidinia; Pulakat and Sumners, 2020; H.; Wu, Wen, Jiang, Liu, & Nie, 2018). ROS complications and oxidative stress lead to the development of unhealthy reactions at the cellular and molecular levels, such as DNA damage, lipid peroxidation, protein degradation, and secretion of antioxidant enzymes. (Kaneto, Katakami, Matsuhisa, & Matsuoka, 2010; Chattopadhyay et al., 2015; Dong et al., 2016a; Panigrahy, Bhatt, & Kumar, 2017). A study reported a relationship between insulin resistance and ROS generation enhancement triggered by treating the pancreatic cells with oxidative stress inducers (Burgos-Moron et al., 2019; De Marañón et al., 2020a; Kassab & Piwowar, 2012; P.; Wang et al., 2018; Wright, Scism-Bacon, & Glass, 2006). Surprisingly, they denoted the cellular events related to ROS enhancement and decreasing the expression of insulin mRNA in the insulin gene (Opara, 2004; Belia et al., 2009; Gonzalez et al., 2011; Yanagi, Monden, Ikeda, Matsumura, & Kasai, 2011). Their studies exploring ROS production inside the cells are strongly involved in insulin resistance in hyperglycemic conditions. In other experimental conditions, long-term exposure of the cells and animals to hyperglycemic conditions could develop insulin resistance, which leads to excess production of ROS (Afanas’ev, 2010; Brown, Bridge, & Kearney, 2021; Mackenzie et al., 2009; Sukotjo, Sargowo, & Wihastuti, 2014). Oxidative stress is known to activate enzymes, such as superoxide dismutase (SOD), glutathione (GPX), catalase (CAT), condensed glutathione, and reduced glutathione (Chang & Chuang, 2010; J.; Chen et al., 2017; Eckers, Altschmied, & Haendeler, 2012). In type 2 DM patients, dropping the daily glucose intake could be a useful strategy to reduce the ROS level (Bunpeng et al., 2022; Boriboonhirunsarn, Boriboonhirunsarn, Sawangpanyangkura, and Tansriratanawong; Z. A.; Ma, Zhao, & Turk, 2012; Nemoto et al., 2007). The development of type 2 DM is well associated with dysglycaemia, chronic sustained hyperglycemia, and acute glycemic fluctuations (W. C. Chao et al., 2015; Dave & Kalia, 2007; Gawlik et al., 2016; Gilardini Montani et al., 2016; Isoni et al., 2009; Mahood, Hussein, & Al-Ahmed, 2018).

The relationship between oxidative stress and DNP, development, and progression requires further elucidation by exploring the detection of biomarkers (Hirao et al., 2010; Khan, Banga, Mashal, & Khan, 2011). In previous reports, oxidative stress as measured by the level of malondialdehyde (MDA) was not related to fasting blood levels (Hayashi, Murakami, Yamamoto, Ono, & Onodera, 1997). MDA was detected in DM, which influences the mitochondrial membrane potential (MMP) and may lead to activation of the electron transport chain complex system to further activate significant pathways for the production of ROS (S. C. W. Tang et al., 2010). In addition, nicotinamide adenine dinucleotide phosphate (NADPH) is the standard complex enzymatic system for the generation of ROS, and it seems like a basic source for glucose-induced ROS production in the tissues/cells of DM prototypes (Jersin et al., 2021; Wong et al., 2010). Besides, xanthine oxidase plays a vital role in the production of ROS, which shows an important role in the development of DM and associated complications (Fidler et al., 2017). In some research studies, glucose and its metabolites react with hydrogen peroxide (H_2_O_2_) in the existence of copper (Cu) and iron (Fe) to generate the hydroxyl radicals (.OH) through Fenton-like reaction during auto-oxidation in DM promoting ROS cascade and DM complications (Figure 3) (Gadjeva, Goycheva, Nikolova, & Zheleva, 2017; Guichard, Moreau, Pessayre, Epperson, & Krause, 2008; J. W. ; Kim et al., 2016).

**FIGURE 4:**
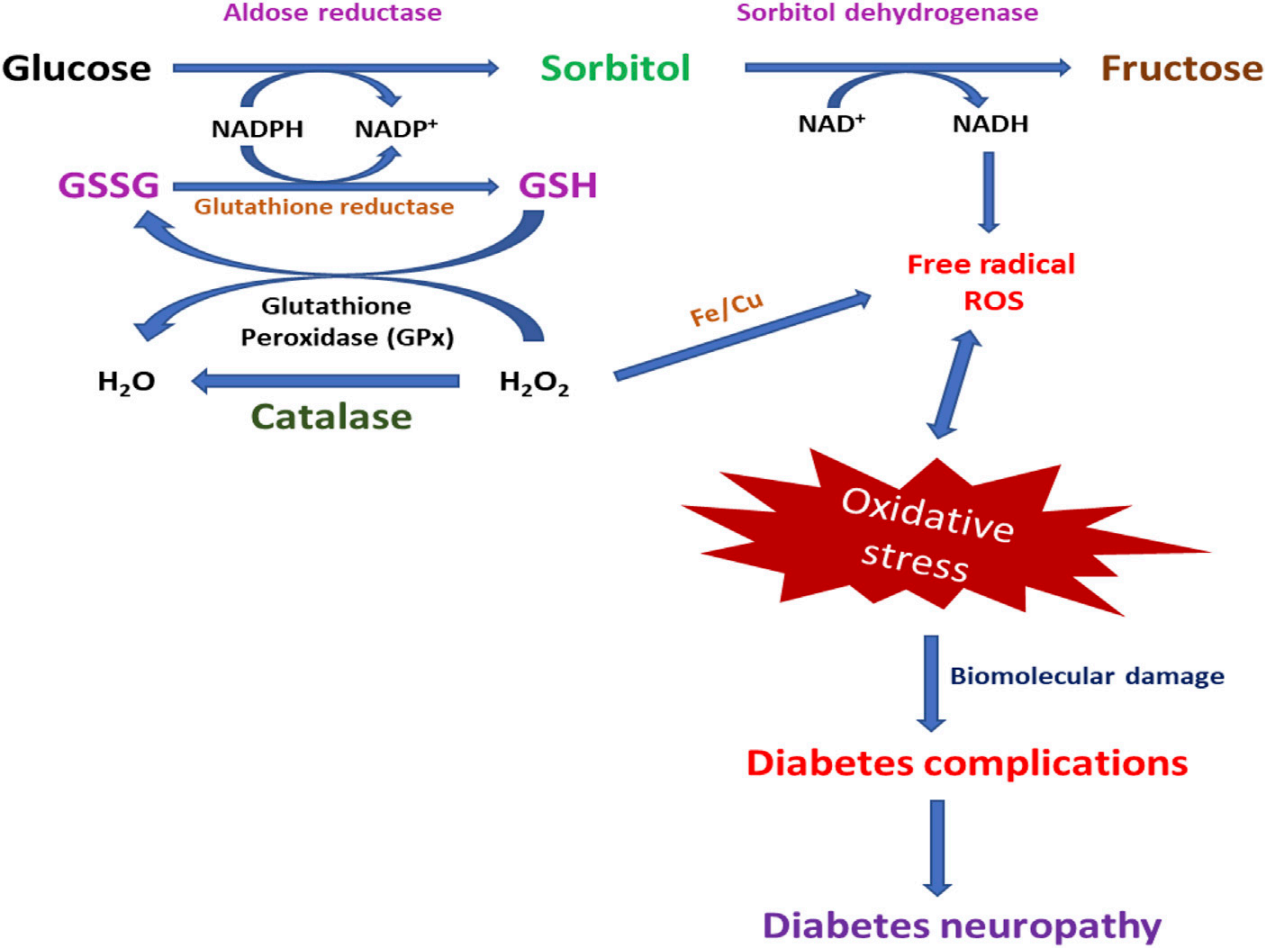
Photographical demonstration of mechanisms of oxidative stress-induced DNP. Oxidative stress-activated pathways for the generation of DNP.

In DM, ROS generation depends on various routes, such as the enhanced formation of advanced glycation end products (AGEs), heightened polyol pathway, and stimulation of protein kinase C (PKC) (Kassab & Piwowar, 2012; Nogueira-Machado et al., 2006). The aldose reductase pathway is a NADPH-dependent enzyme that catalyzes the reduction of glucose into sorbitol (polyol) and goes along with the oxidation of sorbitol to fructose by NAD^+^-dependent polyol dehydrogenase (Eckers et al., 2012; L. He & Sun, 2021). It is assumed that hyperglycemia in the saturation of hexokinase amounts to over 30% of glucose transferred into the polyol pathway (Hamada, Fujii, & Fukagawa, 2009; Wan et al., 2019). The polyol pathway results from lacking intracellular NADPH and an excess of NADH, which generates the NADH oxidase to produce ROS, causing DNA damage (Babizhayev et al., 2015; Takamura et al., 2008). The polyol pathway works as the most important source of ROS generation in the neuron, retina, and nephron, and sorbitol increase concentration has been associated with neuropathy, retinopathy, and nephropathy of DM (Figure 4) (Eckers et al., 2012; Ojima, Matsui, Maeda, Takeuchi, & Yamagishi, 2012; Ferlita et al., 2019; Darenskaya, Kolesnikova, & Kolesnikov, 2021).

AGE development glucose can delightedly act in response with free amino groups of protein to form Schiff bases. These Schiff bases go through complex reactions to form advanced AGEs, which cause tissue destruction through the formation of cross-links that alter the protein structure and function and relations with cell surface receptors on endothelial cells and macrophages, which contributes to the activation of cell signaling and gene expression that induce oxidative stress and inflammation in DM (De Oliveira, Colette, Monnier, Descomps, & Pares-Herbute, 2005; Spadaccio et al., 2014; Dai, Chen, & Chai, 2019).

PKC-dependent stimulation of NADPH oxidase by high glucose levels can promote ROS formation in aortic endothelial cells, smooth muscle, and renal mesangial cells. Laboratory evidence indicates the NADPH oxidase-dependent production of ROS in DNP. Some reports, explained the detailed relationship between retinopathy and DNP by increased levels of NADPH, which leads to the generation of ROS and causes retinopathy and neuropathic complications in DM (Rolo & Palmeira, 2006; Kizub, Klymenko, & Soloviev, 2014; Karunakaran et al., 2015; Panigrahy et al., 2017). Relationships among PKC, NADPH, and ROS generation through different ways in DM are explained through possible examples (M. H. Kim et al., 2017; H. X. Wang et al., 2013). The activation of endothelial nitric oxide (eNOS), NADPH oxidase, vascular endothelial growth factor (VEGF), endothelin-1 (ET-1), transforming growth factor-β (TGF-β), phospholipase A2 (PLA2), and nuclear factor kappa beta (NF-κB) leads to the generation of ROS in DM (F. He et al., 2021; Yao et al., 2021). In another research, diacylglycerol-dependent stimulation of PKC affects the gene expression of key proteins related to blood flow, capillary occlusion, inflammation, and damage of cellular macromolecules in DM. The DM-associated complications, such as high glucose levels, can stimulate ROS generation through PKC and NADPH-dependent pathways in various cells, such as aortic endothelial cells, smooth muscle cells, and renal mesangial cells (N. Jiao et al., 2020; Toma, Sanda, Deleanu, Stancu, & Sima, 2016). In the phagocytic cells, the NADPH oxidase-dependent pathway plays as primary enzyme compared with non-phagocytic cells (Guichard et al., 2008; Sedeek et al., 2010; Kassan et al., 2014). In another research, NADPH oxidase levels in DM animal models are compared with non-DM animals as an indicator of the neuropathy and coronary artery disease treatments (Fagundes-Netto et al., 2013; H. X.; Wang et al., 2013; Xing et al., 2017). Recent studies have shown a relationship between the NADPH-dependent production of ROS in DM, which leads to neuropathy and further consequences (San Martin et al., 2007; Sedeek et al., 2010; Dong et al., 2016b). The activation of antioxidant enzyme production in high glucose levels in DM causes oxidative stress. At cellular levels, we detected high levels of polyunsaturated fatty acids, ferrous ions, and molecular oxygen in reported scientific reports as huge problems in DM. The high levels of glucose and oxidative stress complications play a significant role in the DNP progression and its further complications. Cells have different protection systems to prevent or scavenge the overproduction of ROS and oxidative stress, which includes antioxidant enzymes such as SOD, GPX, and CAT, and overcome oxidative stress and prolongs cell surveillance and participates in the cellular signaling pathways for recovery from DNP (Afanas’ev, 2010; J.; Jiao et al., 2012; Y. H.; Ma, Li, Yin, & Li, 2015; Tseng, Vong, Kwan, Lee, & Hoi, 2016; Yuan et al., 2010).

SOD participates in scavenging the superoxide radical by promoting its conversion to H_2_O_2_, while GPX detoxifies H_2_O_2_ and lipid peroxides (Song et al., 2018; Liao et al., 2019; Dworzanski et al., 2020). Another enzyme, CAT, accelerates the decomposition of H_2_O_2_ into water and molecular oxygen (Yan, Chen, & Zheng, 2017; B. B.; Zhao et al., 2021). Excessive blood glucose can hinder the antioxidant defense system, which influences the actions of antioxidant cascade enzymes in DM conditions (H. Q. Tang et al., 2021; Waldman et al., 2018). In some reports, authors documented changes in the antioxidant defense system in DM as a considerable decline in the SOD enzymatic activity in red blood cells in DM *in vivo* models (Ding et al., 2019; Q.; Lu et al., 2018). The reduction of the SOD activity in DM conditions could be due to oxidative stress-induced inactivation. The enhancement of H_2_O_2_ levels is due to inactivation of SOD, inactivation by glycosylation of SOD, and loss of Cu^2+^ co-factor necessary for the enzymatic activity can be decreased (Bhattamisra, Koh, Lim, Choudhury, & Pandey, 2021; O. H.; Lee et al., 2009; Liao et al., 2019).

**FIGURE 5:**
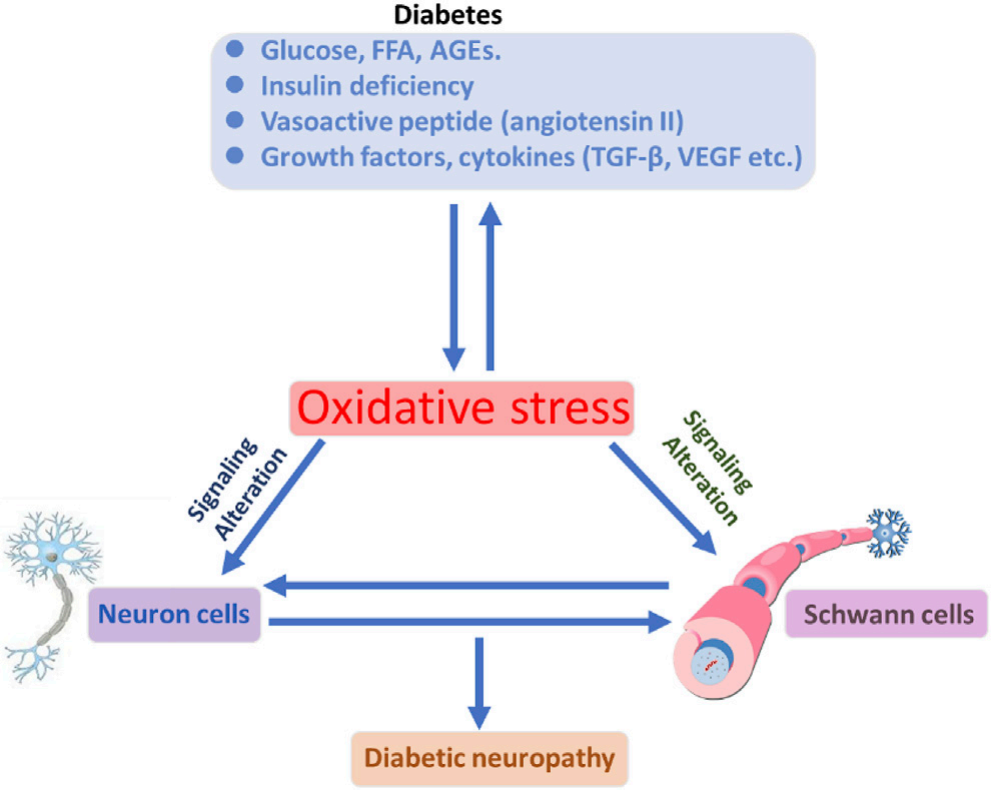
Mechanisms of oxidative stress-induced DNP in injured neuronal cells.

The relationship between oxidative stress and inflammation is an essential physiological response of the body to various disease states, such as pathogen invasion, tissue damage/injury, and irritants. The inflammatory response affects the cell’s infiltration and subsequent activation of the cell’s innate and active immune system at the site of injury by secreting inflammatory mediators’ cytokines (Bagi, Feher, & Beleznai, 2009; J. Wang & Guo, 2019). The scientific reports provide evidence that enhanced the trigger of inflammatory mediators in DM conditions due to high levels of glucose-negotiated oxidative stress. The DM pathophysiology is commonly associated with chronic inflammation and oxidative stress. Complex interactions with these pathways consist of positive feedback mechanisms for both mutual expansions (Figure 5) (Strollo et al., 2013; Dong et al., 2016a). Inflammation is the key factor for the immune responses to eradicate pathogens from the cells and repair the cells to a normal state. Innate immune cells produce pro-inflammatory cytokines and chemokines that promote the production of ROS and RNS. Pro-inflammatory cytokines can ultimately aggravate oxidative stress by encouraging macrophages, which are known to play a key role in eliminating the pathogen *via* the production of ROS (De Marañón et al., 2020b; P.; Wang et al., 2018). It is significant to note that prolonged inflammation is a pathological condition established by tissue damage, fibrosis, and cell damage. The inflammatory conditions contribute and produce their cellular side effects through the generation of ROS, reduction in antioxidants, and enhanced stress-activated kinases (de Maranon, Diaz-Pozo, et al., 2020; Yanagi et al., 2011). At the cellular level, ROS can activate transcription factors such as NF-κB, which influence the secretion of pro-inflammatory cytokines. Under oxidative stress conditions, activation of the immune system commonly has a short life because of intrinsic negative feedback mechanisms such as enhanced generation of the antioxidant cascade system and anti-inflammatory cytokines. Eventually, in chronic DM conditions, activation of the oxidative system and inflammation serve as positive influences to control DM (Drougard et al., 2013; Rovira-Llopis et al., 2013).

Inflammation plays a crucial role in the pathogenesis of type 2 DM in understanding the inflammatory induction factors. According to recent studies, the relationship between type 2 DM and elevation in the levels of sialic acid, C-reactive protein, and interleukin-6 (IL-6) is expected to accelerate the progression of type 2 DM (Belia et al., 2009; Mushtaq, Ali, Altaf, Abdullah, & Murtaza, 2015). Other studies have shown that enhanced levels of inflammatory biomarkers anticipate insulin resistance and progression of type 2 DM (Sukotjo et al., 2014; Brown et al., 2021). In another study, the connection between fasting insulin levels and C-reactive protein (CRP) levels in the plasma shows that insulin resistance and inflammatory methods are connected (Mackenzie et al., 2009; Cantley & Biden, 2013). Nonetheless, the mechanisms of chronic inflammation’s contribution to development of type 2 DM are not clearly understood yet. However, adipose tissue produces important pro-inflammatory cytokines, tumor necrosis factor, and inflammatory biomarkers, which are associated with body fat mass, suggesting that triggered innate immunity and inflammation factors in the pathogenesis of type 2 DM (Caton et al., 2013; Nokkaew et al., 2021).

## Pathogenesis of Diabetic Neuropathy Pain

Similar to the central nervous system (CNS), the PNS is comprised of 12 cranial nerves and 31 pairs of spinal nerves. The PNS is composed of neurons and supporting glial cells (Schwann cells) (Selvarajah, Wilkinson, Griffiths, Emery, & Tesfaye, 2008; Qureshi & Ali, 2021). Commonly, efferent axons from motor neurons transport information from the CNS to muscles and glands. The afferent axons from motor neurons transport information from peripheral sensory receptors to the CNS (El-Salhy, 2002; Kempler et al., 2017). The position of the neuron cell body is also important; sensory neurons, specifically dorsal root ganglion (DRG) neurons, are located outside of the blood–nerve barrier, as do the peripheral sensory receptors, whereas motor neurons are located within the ventral horn of the spinal cord under the protection of the blood–brain barrier (Rondon et al., 2010; Iyer & Tanenberg, 2013).

The DRG neurons are exposed to systemic metabolic and hypoxic stressors, making them much more vulnerable to injury. The anatomy of the sensory system beyond the blood–brain barrier may also explain its exceptional vulnerability (Eaton et al., 2003; Selvarajah, Wilkinson, Gandhi, Griffiths, & Tesfaye, 2011). In the PNS, thin unmyelinated axons known as C fibers carry information for the automatic nervous systems, as well as afferent impulses in response to extreme conditions such as temperature and mechanical force damage to tissue (Mert et al., 2009; Ozkul et al., 2010). Unmyelinated C fibers and small myelinated fibers help with cold detection, which is crucial to the ability to perceive pain, ulcers, and amputation. Simultaneously, developing new ideas into how specific genes, significant pathways, and axonal bioenergy influence injury in DNP, new findings target the pathophysiological processes of causing pain and understanding DNP (Ozkul et al., 2010; Singh, Bansal, Kuhad, Kumar, & Chopra, 2020). Neuropathic pain is a common feature and helps in significantly disabling DNP, pain caused by lesions, or affecting the somatosensory system. The essential unique feature of DNP versus other pain is the paradoxical sensory loss, and with or without the pain sensory hypersensitivity theory, the various sensory modalities in the affected area (Tsuda, Masuda, Tozaki-Saitoh, & Inoue, 2013; Feldman, Nave, Jensen, & Bennett, 2017). The DNP form of pain is different to the chronic inflammatory pain and idiopathic pain where the somatosensory nervous system is undamaged (Inoue & Tsuda, 2009; J. Y.; Zhao et al., 2018). In DM, hyperglycemic conditions promote the various significant pathways in the production of inflammatory cytokines that initially cause neuroinflammation. Later, neuroinflammation conditions show impact on sensory fibers and ion channels, leading to nerve injury and abnormal neuropathic pain.

**FIGURE 6:**
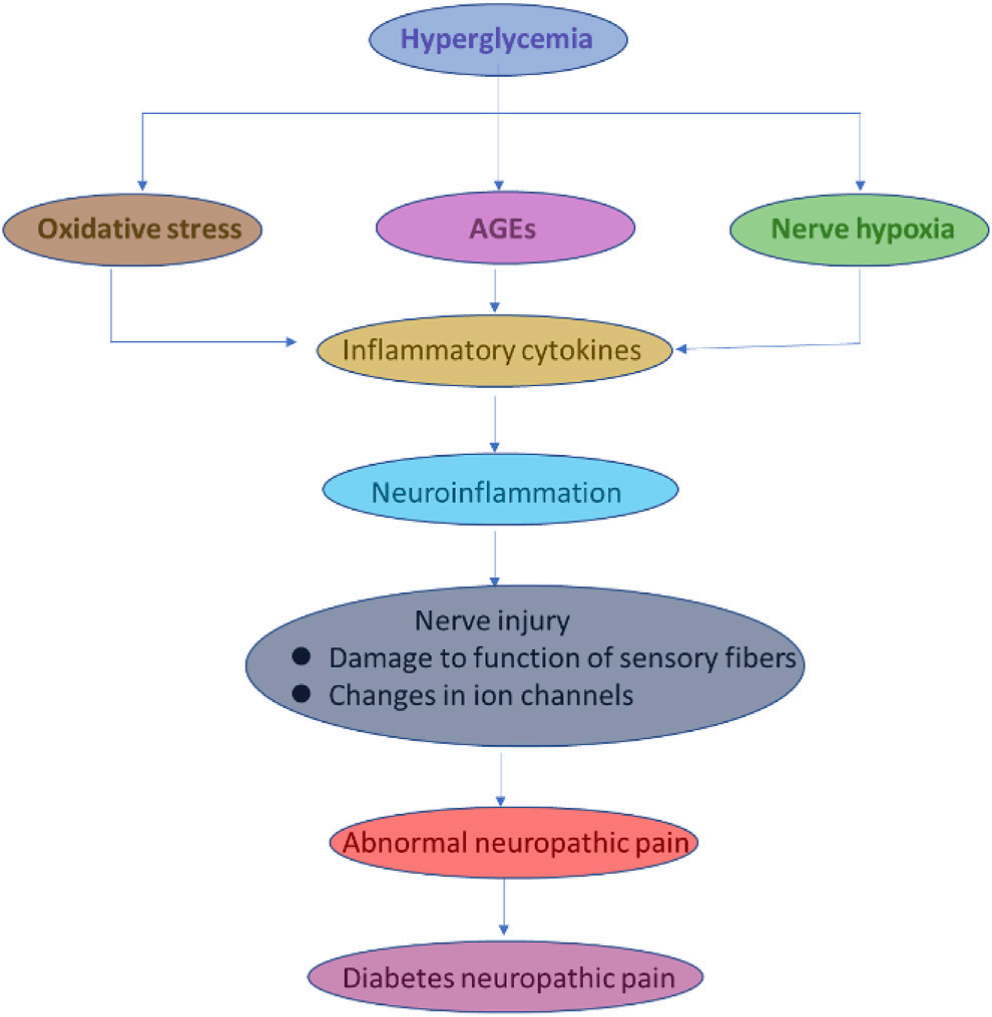
Graphical explanation of pain pathogenesis in DNP.

The signs and symptoms of DNP show sensory loss with less sensitivity to touch, hot and cold temperatures, needle prick, and pain hypersensitivity with hyperalgesia or allodynia (Tavakoli, Mojaddidi, Fadavi, & Malik, 2008). The circulation of symptoms and signs varies on the nerves affected in DNP, in which most common forms are distal symmetric polyneuropathy and pain, and sensory findings are observed in the feet, toe, and occasionally also in the finger of hands (Ambrose & Golightly, 2015; Prajapati, Filippi, & Sears, 2021). Pain has diverse features such as burning, stinging, and shooting. Deep aching types are the most common ones within the region of damaged nerve fibers. This problem remains unsolved as to why some patients develop NP and others remain without pain, even if it is likely to relate to complex interactions between the genotype and environment. Recent two studies demonstrated the significant link between the heritability and environmental factors role. Also, respective huge genome-wide linked studies aids in exploring the relationship between the genotype and occurring DNP (Tsuda, Inoue, & Salter, 2005; Tsuda, 2019). Even though neuronal hyperexcitability is a primary factor in DNP conditions, the clinical studies showing such hyperexcitability are not always present in DNP. In recent studies, relating discussions of patients with clinical examinations in T2DM explains a phenotypic multiplicity in patients with and without pain. Their studies explain that increased sensitivity to thermal and mechanical stimuli is a good selective feature between DNP patients with and without neuropathic pain (Tavakoli & Malik, 2008; Suryavanshi & Kulkarni, 2017). It is convincing that the predominance of sensory loss versus the presence of hyperalgesia and allodynia may explain the failure to find clear characteristic qualities in painful versus painless DNP (Obrosova, 2009; Afrazi & Esmaeili-Mahani, 2014) (Figure 6).

Psychological issues are also critical features to the development of DNP and initially may cause damage to peripheral and brain networks’ subserving pain. Its variations fluctuate among individuals and are expected to have a significant part in the appearance of pain (Q. X. Hong, Xu, Dai, & Zhao, 2016; Tesfaye & Selvarajah, 2012). Some studies show a positive correlation between both neuropathy harshness and poor glycemic control with both the consequence and intensity of DNP pain, relation with genetic predisposition that accurate ion channel variants may be inclined to the progression of painful DNP (P. P. Lu et al., 2020; Lukic, Humpert, Nawroth, & Bierhaus, 2008). Sensory neuron hyperexcitability in the form of impulsive movement and altered incentive response purpose is reported both in patients and in experimental models of DNP. Sensory neuron excitability is administered by the design of expression, trafficking, and function of liganded voltage-gated ion channels. The ion channels are critical for the preliminary transduction of sensory stimuli, action potential production, and propagation, and finally, all neurotransmitters release within the dorsal horn of the spinal cord (Becker, Benromano, Shahar, Nevo, & Pick, 2014; Daugherty, Marquez, Calcutt, & Schubert, 2018). The selective appearance of many of these ion channels by sensory neurons means that they are important for analgesic progress. Painful DNP reveals common pathophysiological mechanisms with other types of neuropathic pains for dysregulation of ion channel manifestation (Kashyap & Farrugia, 2011; Park, Kang, Jeon, Kim, & Lee, 2019).

The cellular significance of the central application of the peripheral neuronal activity is generally displayed by an overstated response to synaptic inputs, a reduction in the threshold to activate neurons, an augmented response to suprathreshold stimuli, and an extension of accessible fields (Alaverdyan & Vartanyan, 2012; Deli, Bosnyak, Pusch, Komoly, & Feher, 2013). Previous reports suggested that these central mechanisms can impact on painful DNP; streptozocin (STZ) rats’ extensive dynamic range of dorsal horn neurons show Rac-1-mediated dendritic spine morphology that is related to natural activity and hyperexcitability to peripheral stimuli (Gao et al., 2019; X. H.; Ma et al., 2020). The reversal of these fluctuations using a Rac-1 inhibitor blocks hypersensitivity both at the electrophysiological and behavioral level. The dorsal horn of the spinal cord is below the control of a descending pain modulatory system that can either inhibit or enable transmission of nociceptive information (Johnson, Ryals, & Wright, 2007; X. Q.; Ma et al., 2019). At the clinical level, conditioned pain modulation is a technique that is used to test these descending controls; importantly, habituated pain variation is reduced in some patients with DNP (Fischer, Tan, & Waxman, 2009). Unusual signaling between neurons and glia also plays a role in pain in the framework of maladaptive plasticity. Following interrupting nerve injury, microglia within the dorsal horn of the spinal cord release factors such as the brain-derived neurotrophic factor (BDNF), which results in the augmentation of nociceptive synaptic processing and thus gating of neuropathic pain (Paulson, Wiley, & Morrow, 2007). Although DNP is related to a gentler rate of de-afforestation associated with traumatic neuropathy, there is also a suggestion for a role of microglia in the progression of neuropathic pain. A significant deliberation is that recent studies report that microglia are essential for the development of powered pain hypersensitivity, following traumatic nerve injury in male but not female rats (Johnson et al., 2007; X. Q.; Ma et al., 2019). In other reports, gender-specific effects have not been studied in the experimental models of painful DNP and highlight the importance of gender when exploring pathophysiological pain mechanisms. Oligodendrocytes have also been associated with showing a role in the central pain mechanism; loss of oligodendrocytes in the spinal cord leads to both an excessive pain response and axon loss in the spinal cord dorsal horn with injury to the spinothalamic tract. Currently, unknown damage to oligodendrocytes in the CNS also contributes to pain in DNP. Recent studies suggest that separate CNS mechanisms independent of a peripherally determined central sensitization may be complicated in pain, following glial damage (Taliyan & Sharma, 2012; Byrne, Cheetham, Vickers, & Chapman, 2015; Yamashita et al., 2019; Jiang, Chen, Chen, Jiao, & Wang, 2021).

## Dipeptidyl Peptidase IV Inhibitors and Glucagon-Like Receptor Agonists in Diabetic Neuropathy Management

**FIGURE 7:**
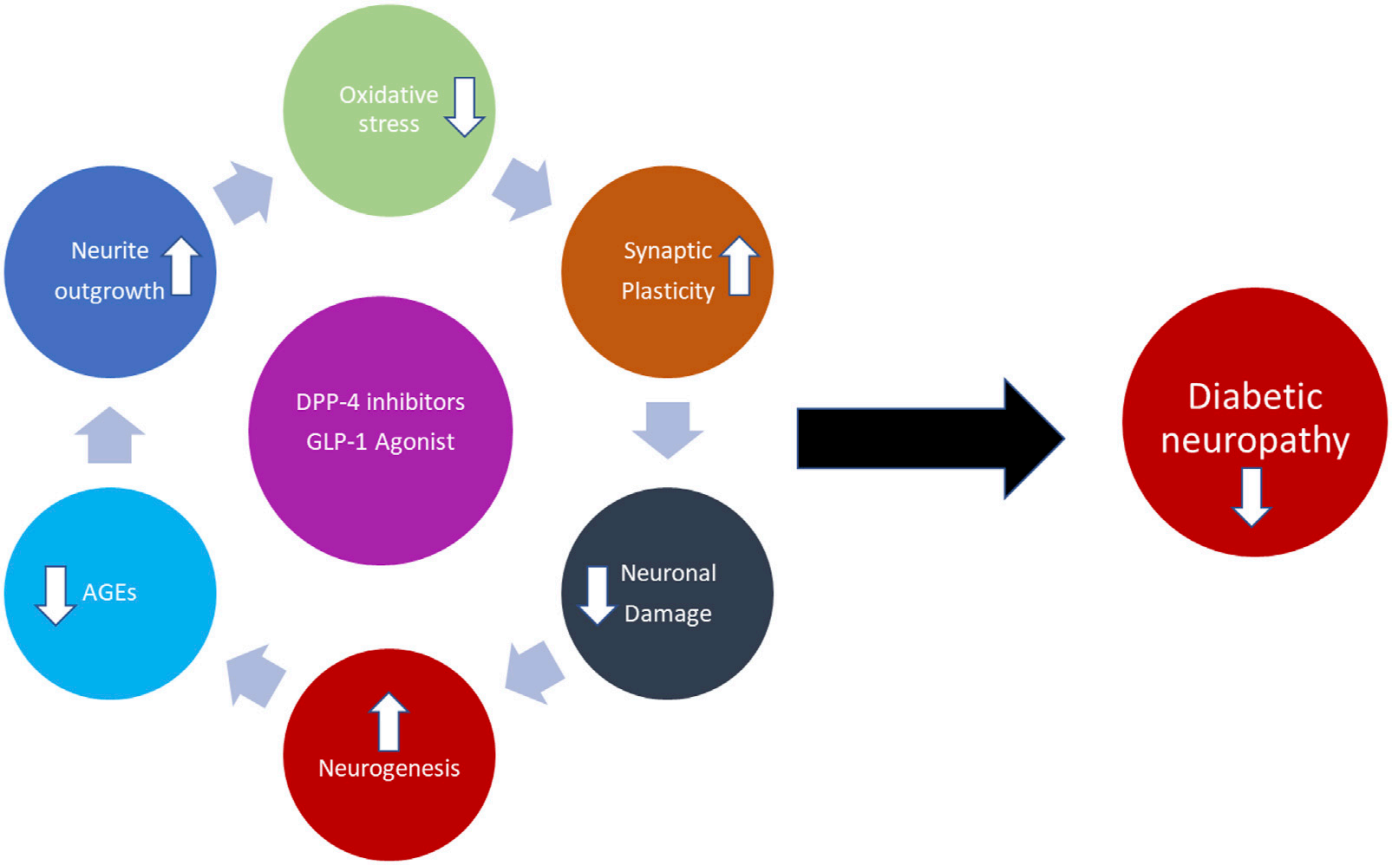
Schematic illustration of the DNP protective effect of the GLP-1 agonist and DPP-4i.

DPP-4 inhibitors are a class of oral hypoglycemics that block the enzyme DPP-4 for treating diabetes. Blocking DPP-4 activates the stimulators of pancreatic insulin secretion, thereby decreasing blood glucose levels (Rosenstock et al., 2006; Jackiewicz & Katarzynska, 2018). On the other hand, increasing studies support the idea that DPP-4 might also be involved in the development of neurological disorders with a neuroinflammatory component, potentially through its non-incretin activities and its inhibition, which is shown to have protective effects on central and peripheral neuropathies such as Parkinson’s disease, Alzheimer’s disease, and DNP (Gong et al., 2014; Civantos et al., 2017). DPP-4i inhibitors have also shown a promising potential in the alleviation of kidney, brain, and heart diseases through their antioxidant functions. Some recent studies have shown that DPP-4i is expressed in the spinal cord and brain regions during neuropathic pain, which is known to activate mitogen-activated protein kinase (MAPK) pathways by ROS generation (Jing, Zou, Wang, Cai, & Tang, 2021; C. H.; Lee et al., 2018). Its inhibition alleviated hyperalgesia through the selective blocking of glial cell activation. Apart from the deactivation of glial cells, DPP-4 inhibition with drugs such as tripeptide isoleucin-prolin-isoleucin, alogliptin, and the antidiabetic drug vildagliptin is reported to possess antihyperalgesic effects through opioid-dependent and opioid-independent mechanisms in inflammatory and neuropathic pain animal models (Xu et al., 2017; B.; Zhu et al., 2014). Oral administration of DPP-4i such as teneligliptin (TEN) is known to possess analgesic effects in humans against thermal pain. In addition, TEN is also known to enhance glutathione antioxidant production within the cells (Fan et al., 2015; Yoon, Kim, & Song, 2020; Kuthati, Rao, Busa, & Wong, 2021). Recently, several reports have concluded that activation of GLP-1R can suppress neuroinflammation and central sensitization, thereby attenuating neuropathic pain in animal models (Xu et al., 2017; B.; Zhu et al., 2014). Furthermore, in the CNS, GLP-1R is constrained to the microglial cells of the dorsal horn (Jing et al., 2021). The GLP-1R expression is known to decrease after nerve injury and is one of the important factors in microglial cell activation (C. H. Lee et al., 2018). Exenatide, a GLP-1R agonist, is effective in the alleviation of oxaliplatin-induced peripheral neuropathy. The analgesic properties were reversed by either the administration of GLP-1 antagonists or GLP-1 gene knockdown in the animal models of neuropathic pain (Gong et al., 2014). Exendin-4, an agonist of GLP-1, alleviated pain-induced cognitive impairment by suppressing neuroinflammation in neuropathic pain rats (Cui, Fong, Zhang, Xia, & Zhan, 2020). Additionally, DPP-4i such as sitagliptin and GLP-1 analog liraglutide are shown to induce axonal regrowth and locomotor functional repair through the restoration of spinal GLP-1R levels in SCI rats (Han et al., 2020). Recently, we have reported the analgesic effects of DPP-4 TEN against partial sciatic nerve transection–induced NP through the suppression of spinal astrocytes and restoration of spinal GLP-1R (Nakashima, Kaneto, Shimoda, Kimura, & Kaku, 2018). Though several studies have reported the efficacy of DPP-I’s and GLP-1R’s in neuropathic pain animal models using sciatic nerve injury, there are very few studies that have analyzed the efficacy of these compounds in diabetic neuropathic pain animal models (Han et al., 2020). DM conditions have various cellular environments, such as enhanced oxidative stress, AGEs, neuronal damage conditions, and decreased neuronal growth, synaptic plasticity, and neurogenesis. The DPP-4i and GLP-1 used in the treatment reversed all those conditions and reduced diabetes neuropathy and pain. There is a need to pursue future research in this direction to determine if this class of drugs has any beneficial effects over other types of diabetic drugs for DNP management (Figure 7) (H. Y. Wu, Mao, Fan, & Wang, 2017).

## Melatonin and its Functions in Diabetic Neuropathy

Melatonin is a broad-spectrum antioxidant with remarkable therapeutic effects against ROS-related diseases through the regulation of oxidative stress (X. X. Zhang et al., 2020). Melatonin has an easier access to subcellular organelles such as the mitochondria, the primary sites for ROS production due to their lipophilic and hydrophilic characteristics (Reiter et al., 2003; Kuthati, Busa, Davuluri, & Wong, 2019). Melatonin can induce antioxidant effects either through direct free radical scavenging or through the synthesis of antioxidant enzymes (Posa, De Gregorio, Gobbi, & Comai, 2018). Melatonin has possible therapeutic effects for treating neurodegenerative diseases, cardiovascular diseases, and many other clinical complications (Amer, Othamn, & El-Missiry, 2021). Melatonin also shows immunomodulatory consequences which depend on its ability to augment the cytokine levels and antioxidant properties (Edizer et al., 2019; Kuthati et al., 2020).

It is estimated that 50% of diabetic patients suffer from DNP, with symptoms involving pain, foot ulcers, and amputation. Oxidative stress and neuroinflammation are known to play an important role in the pathogenesis of DNP (Liang et al., 2020). The high prevalence of DNP in diabetic patients results from multiple reasons. First, diabetic patients have enormous demand for brain oxygen and polyunsaturated fatty acids (PUFAs), resulting in incompetent antioxidant capability within the brain, making it vulnerable to ROS (Onphachanh et al., 2017). In addition, hyperglycemia initiates hippocampal and cortical neuronal degeneration, leading to amputation. DM is also known to damage somatic and autonomous nervous systems, leading to DNP and diminishing life quality.

## Effect of Melatonin Treatment on Diabetic Neuropathy in Experimental Findings

As oxidative stress plays an important role in the initiation and progression of DNP; melatonin can be considered as an effective adjuvant in the management of DNP (J. L. Zhang, Hui, Zhou, & Hou, 2018). Melatonin administration is shown to inhibit DNP symptoms through the increase of motor nerve conduction velocity (MNCV) and sciatic nerve diameter (J. L. Zhang et al., 2018). The antihyperalgesic effects of melatonin are medicated through the inhibition of the L-arginine–NO pathway (Kandemir, Guntekin, Tosun, Korucuk, & Bozdemir, 2018). In addition, the analgesic effects of melatonin are corroborated by the melatonin type 2 (MT2) receptor actions through the modulation of brainstem descending pathways (Posa et al., 2018). Melatonin is known to ameliorate hippocampal injury and DNP in STZ-induced diabetic rats. Melatonin is known to alleviate oxidative stress-triggered calcium signaling through the expression of transient receptor potential (TRP), melastatin 2 (TRPM2), and TRP vanilloid type 1 (TRPV1) in the hippocampus (Kahya, Naziroglu, & Ovey, 2017). Melatonin is also known to minimize myelin sheath vacuolization, separation of axon-myelin sheaths, and degeneration of fibers. Though melatonin does not affect the insulin levels directly in DNP rats, it is known to increase brain antioxidant levels and potentiate the beneficial effects of insulin in DNP rats (Gurel-Gokmen et al., 2018; Liang et al., 2020).

The modulative effects of melatonin on inflammation and autophagy are one of the significant mechanisms through which melatonin alleviates DNP (Jangra, Datusalia, Khandwe, & Sharma, 2013). Melatonin promotes the expression of nuclear factor erythroid-2 related factor 2 (Nrf2), which consequently activates phase II antioxidant enzymes such as heme oxygenase-1 (HO-1) and offers protection from oxidative damage (Ahmadi & Ashrafizadeh, 2020; Aliyev, Panieri, Stepanic, Gurer-Orhan, & Saso, 2021). Melatonin is also known to stimulate PTEN-induced putative kinase 1 (PINK1 expression) *via* an MT2/Akt/NF-κB pathway. The activation of PINK1 is known to aid in the prevention of neuronal cell death under high glucose conditions (Onphachanh et al., 2017). Melatonin is also known to ameliorate diabetes-induced erectile dysfunction in rats through the reduction of oxidative stress and p38/MAPK levels (Sotolongo, Ghiso, & Rostagno, 2020). Additionally, melatonin is also shown to be effective in alleviating diabetes-induced retinal neuropathy through the upregulation of glutamate cysteine ligase in DNP rats through the activation of Nrf2 in the nucleus and stimulation of Akt phosphorylation (Shieh, Wu, Cheng, & Cheng, 2009). In addition, melatonin treatment has also been shown to inhibit the production of pro-inflammatory cytokines through the NF-κB pathway (Tiong, Ng, Koh, Ponnudurai, & Chye, 2019; Nopparat et al., 2021).

One of the most commonly used models for DM is STZ injection in animals (O'Brien et al., 2018). STZ is an antibiotic generated by *Streptomyces achromogenes* that selectively damages the pancreatic β cells (Bour-Jordan et al., 2013; O'Brien, Sakowski, & Feldman, 2014), leading to hypoinsulinemia and hyperglycemia (Ozaki, Terayama, & Matsuura, 2018). The existing literature suggests that DM induces changes in the CNS and PNS, such as DRG and superior cervical ganglion (SCG). Furthermore, it is also known to inhibit the Na^+^K^+^ ATPase pump activity (Pham, Matsumura, Katano, Funatsu, & Ito, 2019). DM is also known to decrease the total area of the pineal gland and reduce the diameter of pinealocytes (Sevak & Goyal, 1996; Yadav, Nagori, & Desai, 2014). Some studies have reported the drastic reduction of pineal melatonin levels after DM induction through pinealocyte alteration (Sevak & Goyal, 1996; Yadav et al., 2014). In contrast, other studies have reported the reduction of melatonin through hyperglycemic signaling and Na^+^_-_K^+^ ATPase activity without the alteration of pinealocytes in DM rats (Pillai, Subramanian, & Kandaswamy, 2013).

Several studies have described the effects of melatonin treatment on DM and DNP using different approaches. Melatonin is known to suppress the upregulation of oxygen and nitrogen free radicals in DM (Adeghate, Rashed, Rajbandari, & Singh, 2006; Silva-dos-Santos et al., 2020). Melatonin is also known to suppress the overexpression of DM-induced inflammatory biomarkers’ levels including TNF-α, IL-6, iNOS, and leptin (Adeghate et al., 2006; Silva-dos-Santos et al., 2020).

DM is known to damage various regions of the brain, including the hippocampus, cortex, and cerebellum, due to the excessive oxidative stress (do Prado et al., 2020; Tong & Cheng, 2005). Melatonin is shown to protect the brain from DM-stimulated glial cell activation and ROS in STZ-induced diabetic rats (Barber et al., 2000, Antonetti, Gardner, and Penn State Retina Res, 2000; Renno, Alkhalaf, Afsari, Abd-El-Basset, & Mousa, 2008) (La Morgia et al., 2010; Galley et al., 2017). In another study, diabetes induction in astrocyte cell culture with high glucose supplementation resulted in an enhanced expression of cytokines and ROS. Addition of melatonin to the cell culture medium resulted in the attenuation of cytokine and ROS levels (Maher, Saleh, Elguindy, Hashem, & Yacout, 2020). One recent study explored the effect of melatonin on central neuropathy by inducing DM through STZ injection in Wistar rats. DM rats displayed neurodegeneration, microglial, and astrocyte activation. Treatment with melatonin abrogated these effects, although melatonin alone did not show any hypoglycemic effect in DM rats (Metwally, Ebraheim, & Galal, 2018). Tiong et al. investigated the antioxidant properties of melatonin at the mitochondrial level in Schwann cells cultured in high glucose. Melatonin is shown to offer protection from glucose-induced mitochondrial membrane depolarization (Tiong et al., 2019; J.; Wang et al., 2012).

Negi and colleagues investigated the protective effects of melatonin and nicotinamide combinational therapy in DM rats on neuronal function, biochemical, and behavioral parameters. Melatonin alone or in combination with nicotinamide improved DM-induced loss of the motor nerve conduction velocity, sciatic nerve blood flow, and pain sensitivity (Negi, Kumar, Kaundal, Gulati, & Sharma, 2010). Melatonin is also known to halt DM-induced activation of the NF-κB cascade of pro-inflammatory cytokines and cyclooxygenase-2 in the sciatic nerve of Sprague–Dawley rats (Seyit, Degirmenci, & Oguzhanoglu, 2016). Melatonin modulates neuroinflammation by lowering the activation of NF-κB and Nrf2 pathways, which may be responsible for its neuroprotective effects in DNP (Seyit et al., 2016).

Orally administered melatonin is shown to alleviate formalin-induced nociception at a low dose, whereas high doses are reported to have anti-allodynia and anti-hyperalgesic effects in DM rats (Arreola-Espino et al., 2007). Moreover, it was observed that K-185 blocked melatonin’s anti-nociception, while naltrindole and naltrexone lowered the antinociceptive effects of melatonin (Arreola-Espino et al., 2007). Previously, melatonin was not known to interact with opioid receptors; in contrast, Arreola and team reported interactions between melatonin and opioid receptors (Arreola-Espino et al., 2007). DM is known to reduce rotarod performance. Treatment with melatonin is shown to improve the rotarod performance. Neurochemical tests have suggested that DM enhances the levels of MDA, acetylcholinesterase, and glutamate in the hippocampus, while treatment with melatonin reverses all changes (Che et al., 2020). One recent report concluded that melatonin is effective in the amelioration of DM-induced PNS neuropathy in Sprague–Dawley rats. The author reported augmented deposition of collagen, oxidative stress, and phosphorylated p38 protein in DNP rats, which were reversed by melatonin (Areti, Komirishetty, Akuthota, Malik, & Kumar, 2017).

Melatonin alone or in combination with drugs such as gabapentin enhanced the hepatic gene expression of peroxisome proliferator–activated receptor and mitochondrial transcription factor-A. Thus, melatonin shows protective effects against DNP in experimental animal studies through multiple pathways (Chahbouni et al., 2017; Juybari et al., 2019). One recent randomized double-blind clinical trial evaluated the potency of melatonin as an adjuvant to pregabalin for pain reduction in painful DNP. Melatonin treatment displayed a decrease in pain sensitivity and a pain-related sleep interference score compared with control (Do Amaral, Andrade-Silva, Kuwabara, & Cipolla-Neto, 2019; Shokri, Sajedi, Mohammadi, & Mehrpooya, 2021).

## Conclusion

This comprehensive review gives a full insight into DM and its complications with detailed molecular mechanisms, pathogenesis, and the ROS generation pathways. DNP and neuropathic pain are the most prevalent complications in DM patients, which is a challenging socioeconomic burden to the world. ROS plays a vital role in the oxidative stress at a cytopathic level, leading to the progression of DNP and neuropathic pain. Gaining knowledge regarding the molecular and biochemical mechanisms of abnormalities related to oxidative stress’s role in DNP and neuropathic pain is essential for the development of new pharmaceutical agents. We have explained various metabolic abnormalities caused by hyperlipidemia, hyperglycemia, polyol signaling pathway, AGEs formation, PKC pathway, and hemosome pathway for enhanced generation of ROS, which subsequently accelerates the oxidative stress in DNP and neuropathic pain. Oxidative stress potentiates the abnormalities of these metabolic pathways, which impact cell mitochondrial membrane damage, neuronal cell damage, lipid peroxidation, and irregular genetic modification of genes in the antioxidant defense system, causing pain and damage to neuronal function. We believe that all the topics discussed in this review, with clear mechanisms and pathogenesis roles of oxidative stress in the progression of DNP, may be promising subjects for further studies on DNP, which open up new platforms for the proper management of DNP and neuropathic pain. Therefore, the inhibition of ROS generation and the evacuation of excessive ROS in neuronal cells could protect the neuronal cells from hyperglycemia-mediated oxidative stress impairments. Due to the nature of the antioxidant properties of melatonin and DPP-4i drugs, these drugs could be ideal for the treatment of DNP and neuropathic pain complications with various mechanisms of action. We explained various mechanisms through which melatonin and DPP-4i drugs can alleviate DNP and neuropathic pain. According to our knowledge, very little research was conducted on DM, DNP, and neuropathic pain using melatonin and DPP-4i. We strongly suggest future researchers focus their research on these drugs alone or in combination with other drugs for the management of DM to limit the adverse effects. In our opinion, even though all *in vitro* and *in vivo* studies provide useful information and hints for clinical trials with melatonin and DPP-4i or its combinational use, extensive work in clinical studies is still essential, and it may be possible to employ multi-functional drugs with a different mechanism of action to fight these multifactorial complications in DM of DNP and neuropathic pain.

## Abbreviations

AGES, advanced glycation end products; BDNF, brain-derived neurotrophic factor; Cu, copper; CAT, catalase; CRP, C-reactive protein; CNS, central nervous system; DM, diabetes mellitus; DPP-4i, dipeptidyl peptidase IV inhibitors; DRG, dorsal root ganglion; DNP, diabetes neuropathy; eNOS, endothelial nitric oxide; ET-1, endothelin-1; Fe, iron; GLP-1R, glucagon-like receptor agonists; GPX, glutathione; GFAP, glial fibrillary acidic protein; OH, hydroxyl radicals; H_2_O_2_, hydrogen peroxide; HO-1, heme oxygenase-1; IL-6, interleukin-6; MNCV, motor nerve conduction velocity ; MT2, melatonin type-2 receptor; MMP, mitochondrial membrane potential; MDA, malondialdehyde; MAPK, mitogen-activated protein kinase; NF-kB, nuclear factor kappa beta; Nrf2, nuclear factor erythroid 2- related factor 2; NADPH, nicotinamide adenine dinucleotide phosphate; PNS, peripheral nervous system; PUFAs, polyunsaturated fatty acids; PLA2, phospholipase A2; PKC, protein kinase C; Redox, reductive and oxidative; ROS, reactive oxygen species; RNS, reactive nitrogen species; SOD, superoxide dismutase; STZ, streptozocin; SCG, superior cervical ganglion; TNF-α, tumor necrosis factor–α; TGF-β, transforming growth factor-β; TEN, teneligliptin; TRP, transient receptor potential; TRPM2, melastatin 2; TRPV1, TRP vanilloid type 1; VEGF, vascular endothelial growth factor.
